# Publisher’s Note: Continued Publication of *Clinics and Practice* by MDPI

**DOI:** 10.3390/clinpract11010001

**Published:** 2020-12-04

**Authors:** Shu-Kun Lin, Unai Vicario, Franck Vazquez

**Affiliations:** 1 MDPI, St. Alban-Anlage 66, CH-4052 Basel, Switzerland; lin@mdpi.com; 2 MDPI, Avenida Madrid, 95, 08028 Barcelona, Spain; vicario@mdpi.com

*Clinics and Practice* was launched in 2011 and it has been published over the past nine years by PAGEPress Publications [1]. Dr. Camillo Porta has served as its Editor-in-Chief since its inception [2].

We are delighted to take over the publication of *Clinics and Practice* from PAGEPress and wish to ensure the reader that we will continue to serve health professionals and clinicians well. *Clinics and Practice* complements the MDPI portfolio of medical journals very well [3,4], especially *Journal of Clinical Medicine* [5], *Medicina* [6], and *Medical Sciences* [7], and strengthens the trans-disciplinary bridge between basic sciences and applied sciences across MDPI journals.

We will regularly publish four quarterly issues from 2021.

We hope that you enjoy publishing your work in *Clinics and Practice* [8]!
